# Application of ultrasound in assessment of acute lung injury in mice

**DOI:** 10.1371/journal.pone.0332912

**Published:** 2025-10-07

**Authors:** An-Xia Deng, Ling Zhao, Amanguli Ruze, Qiu-Lin Li, Bang-Hao Zhao, Su Hu, Min-Tao Gai, Hong-Li Wang, Qiao-Ling Yao, Xiao-Ming Gao

**Affiliations:** 1 State Key Laboratory of Pathogenesis, Prevention and Treatment of High Incidence Diseases in Central Asian, Department of Cardiology, The First Affiliated Hospital of Xinjiang Medical University, Clinical Medical Research Institute of Xinjiang Medical University, Urumqi, China; 2 Xinjiang Key Laboratory of Medical Animal Model Research, Urumqi, China; 3 Department of Cardiology, the Fifth Affiliated Hospital of Xinjiang Medical University, Urumqi, China; 4 Department of Physiology, School of Basic Medical College Sciences of Xinjiang Medical University, Urumqi, China; Baylor College of Medicine, UNITED STATES OF AMERICA

## Abstract

This study evaluated the effectiveness of non-invasive ultrasound technology in monitoring pulmonary edema in a mouse model of acute lung injury (ALI). Male C57BL/6J mice were instilled through the trachea with lipopolysaccharide (LPS, 5 mg/kg, single dose) to induce ALI. The change in B-lines (an ultrasound sign) were monitored using the Vevo 3100 system and the lung ultrasound (LUS) score was generated to quantify the severity of edema, meanwhile lung function was assessed by whole-body plethysmography (WBP) and the progression of ALI was tracked by lung weight and histopathological analysis. LUS detection revealed that non-converging B-lines appeared on the first day, followed by an increase in the number of B-lines that began to converge, peaking on the day-3 and then the number of B-lines decreased, almost disappearing by the 11 days after LPS intervention. Significant changes in lung functional parameters were recorded during 6–24 h with most parameters returning to baseline levels at day-3. Morphological and weight assessments of the lung showed that most severe lung congestion and increased weight at day-3 and returning to normal by 11 days. Histopathological examination unveiled that LPS-induced inflammation was characterized by increased cellular infiltration, thickening of alveolar septa, vascular congestion, and atelectasis, which were most severe on days 3–5 and then gradually improved. A positive correlation between LUS score and the lung injury index (*r* = 0.783, *P* < 0.001) or the lung weight-to-tibial length ratio (*r* = 0.695, *P* < 0.001) was observed, reflecting a sensitivity of LUS to the severity of edema. In addition, at 24 h following LPS intervention, the LUS score was also positively or negatively correlated with a series of lung functional indices. In conclusion, the study demonstrated that LUS is an effective, reliable and non-invasive tool for monitoring pulmonary edema in a mouse model of ALI. The significant correlations between LUS scores with various pathophysiological parameters, highlight its potential in assessing the severity of ALI in mice.

## Introduction

Acute lung injury (ALI) is an acute hypoxic respiratory disorder of the lungs [1]. ALI can progress to acute respiratory distress syndrome (ARDS), which is characterized by increased alveolar capillary permeability with acute bilateral pulmonary edema and severe hypoxia [2]. According to the World Health Organization, ARDS affects approximately 10% of intensive care unit patients and has a mortality rate of up to 40% [3]. The pathology of ALI is characterized by severe airway inflammation, disruption of the integrity of epithelial and endothelial barriers and recruitment of neutrophils, T cells and macrophages into the alveolar space [4]. Infiltrating immune cells release a variety of inflammatory and oxidative mediators, which damage endothelial barrier, increase pulmonary capillary permeability and results in excessive accumulation of extravascular lung water, manifested as pulmonary edema [5]. Pulmonary edema severely affects alveolar gas exchange and leads to acute hypoxic respiratory failure [6].

Lipopolysaccharide (LPS) is a commonly used substance for constructing animal models of ALI [7], and as a major component of the outer membrane of Gram-negative bacteria, it can cause various infections, including pneumonia and sepsis [8]. The ALI model induced by intratracheal instillation of LPS is characterized by inflammatory cell infiltration and increased pulmonary capillary permeability, mimicking the pathological features of clinical ALI [9]. The B-line, an lung ultrasound (LUS) sign, is a useful and simple imaging indices for assessing extravascular lung water [10]. Compared to micro-CT and X-ray, LUS has advantages in terms of cost and convenience [11]. LUS is highly regarded for its noninvasive nature, ease of operation, and suitability for long-term monitoring [12]. As an emerging diagnostic technology, LUS is becoming increasingly popular in clinical applications. In recent years, LUS has been used not only in the clinical assessment of patients with acute respiratory failure, circulatory failure, or cardiac arrest [13] but also in large animal models such as pigs [14] and rodent rats [15] with ALI. The pioneering research by Soldati G and colleagues confirmed the effectiveness of ultrasound technology in detecting lung injury. In ex vivo experiment, the researchers manipulated lung tissue density of New Zealand white rabbits by adjusting inflation levels to simulate various physiological and pathological conditions. They observed that at 17% of the resting chest wall volume, a few vertical B-lines appeared; and when the lung volume was further reduced to 12% of the resting chest wall volume, a dense and compact pattern of B-lines was commonly observed [16]. However, current research using LUS for the detection and diagnosis of pulmonary edema is limited to rats and larger-sized animals, with no reports on the use of this technique to detect pulmonary edema in mice.

The aim of the study is to evaluate noninvasive ultrasound technique in precise detection of pulmonary edema in mice with ALI. Using LUS to detect and characterize changes of “B-lines” during the development of LPS-induced ALI, meanwhile, lung function, weight, histological changes and circulating levels of inflammatory mediators are also measured to validate the usefulness of LUS in assessment of the severity of pulmonary edema.

## Materials and methods

### 1. Animals

Male C57BL/6 mice, 8–10 weeks old and at 24–26 g weight, purchased from the Experiment Animal Center of Xinjiang Medical University (Urumqi, China). Mice were housed in an environment with a temperature of 22 ± 2°C, a relative humidity of 55 ± 5%, and a 12-hour light-dark cycle and had free access to food and water. All animal care and experimental procedures were reviewed and approved by the Animal Ethics Committee of Xinjiang Medical University in China, in compliance with the Principles of Laboratory Animal Care and Use (Ethical Approval Number A240301-146).

### 2. Establishment of murine ALI model

To establish an ALI model, mice were anesthetized with 5% isoflurane (RWD Life Science, China), and then tracheal intubation was performed using a 20G intravenous catheter. Through the intubation, LPS (Escherichia coli O55:B5, Sigma-Aldrich, USA) was slowly and directly instilled into the bronchi at a dosage of 5 mg/kg, with a total volume of 50 μl. Mice subjected to LPS intervention were randomly divided into two groups. The first group (contained 12 mice) underwent longitudinal and multiple lung ultrasound examination from day 0 (baseline), 1, 2, 3, 5, 7, 9, and 11 days following LPS intervention, and lung function test from baseline (0 hour, 0 h), 6, 12, 24, 48, 72, and 120 h after LPS titration. The second group comprised 8 subgroups (each containing 5–6 mice) covering 8 time points, i.e., day 0 (baseline), 1, 2, 3, 5, 7, 9, and 11 following LPS intervention. Mice in each subgroup received lung ultrasound examination first and were then sacrificed by exsanguination under 5% isoflurane anesthesia for lung tissue collection for histological analysis and correlation analysis. During the experiment, appropriate measures were taken to ensure the welfare of the mice, to minimize animal suffering and stress, in compliance with the ethical standards for animal experimentation.

### 3. LUS examination and image analysis

In humans, the 28-rib space technique was used for semi-quantitative assessment of pulmonary edema. The scanning regions include the 2nd to 4th intercostal spaces on the left hemithorax and the 2nd to 5th intercostal spaces on the right hemithorax. Scans are performed at four anatomical locations within each intercostal space: the parasternal line, the midclavicular line, the anterior axillary line and the midaxillary line, resulting in a total of 28 pulmonary ultrasound images acquired from the 7 intercostal spaces (3 × 4 + 4 × 4 = 28). Ultimately, the number of B-lines in these 28 ultrasound images was quantified [12]. However, this measurement is not suitable for the small size of mice, we modified the 28-rib space technique by reducing rib spaces to 16–20. LUS was performed using a high-frequency ultrasound system, Visual Sonics Vevo® 3100 System (Visual Sonics Inc, Canada), with a 40 MHz linear-array transducer.

**Fig 1:**
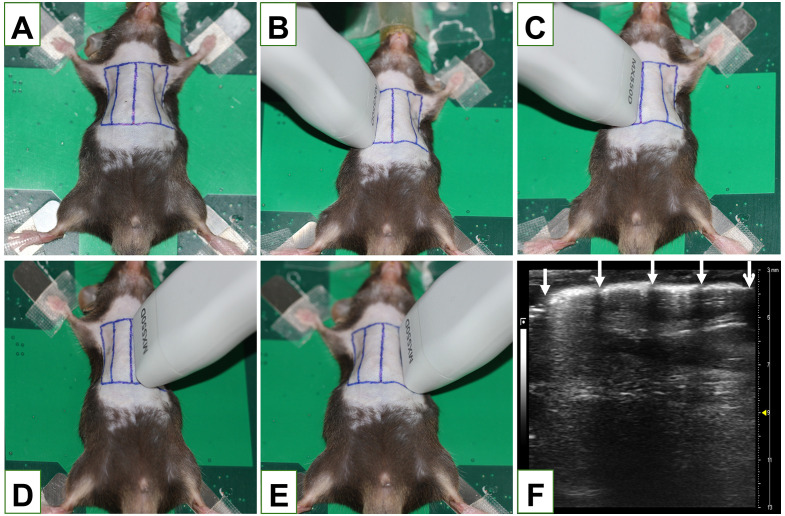
Position of mice undergoing lung ultrasound (LUS) imaging and the areas examined. **(A)** Mice were placed in a supine position to obtain LUS images from the marked four regions on the chest. The scanning order is from **(B)** right anterior axillary line to right midclavicular line; **(C)** right midclavicular line to anterior midline; **(D)** anterior midline to left midclavicular line and to **(E)** left midclavicular line to left anterior axillary line. **(F)** Four to five rib gaps are visible in each scanned area. In the LUS image, the 5 arrows represent rib shadows, and the white line between the arrows represents the pleural line.

**Fig 2:**
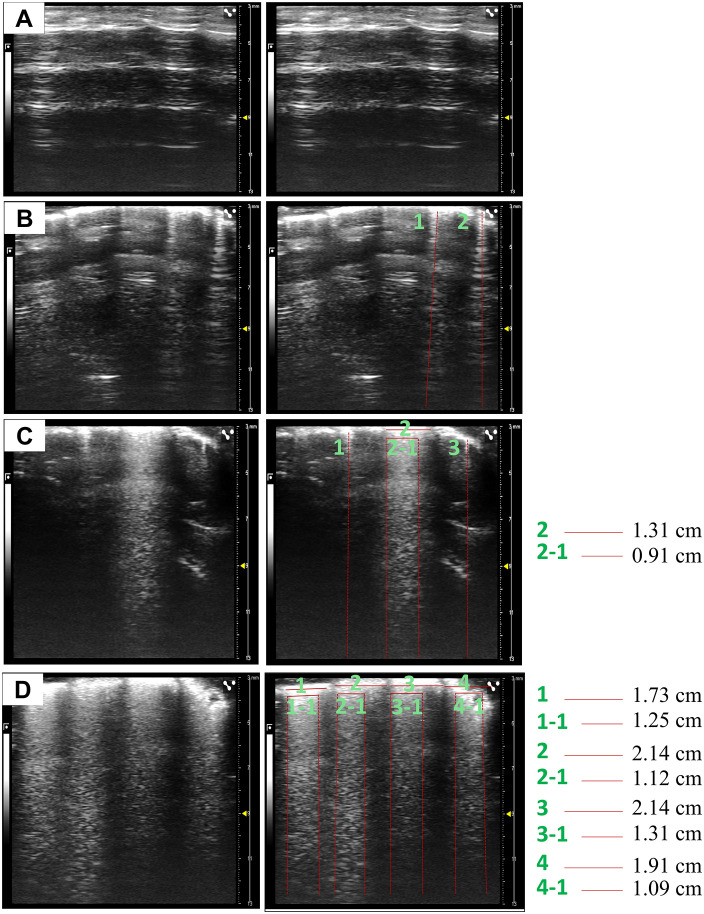
Specific calculation of B-line score. **(A)** In the ultrasound image on the right, only A-lines presented without B-lines, the B-line score for this area = 0. **(B)** As indicated by the red lines, two vertical B-lines (1 and 2) can be seen, with a B-line score of 2 for this area. **(C)** Single and fused B-lines can be observed. B-lines marked as 1 and 3 are not fused, thus B-line score 2. The B-line marked as 2 (2−1) indicates a fused B-line, with a score calculated as (0.91 cm/1.31 cm × 10 = 6.94). The total B-line score for this area is 6.94 + 2 = 8.94. **(D)** B-lines between four intercostal spaces were fused as indicated by red lines and numbered with 1 (1−1), 2 (2−1), 3 (3−1) and 4 (4−1) on the right ultrasound image, the B-line score for this area = (1.25 cm/1.73 cm + 1.12 cm/2.14 cm + 1.31 cm/2.14 cm + 1.09 cm/1.91 cm) × 10 = 24.2. The final B-line score for this mouse were the sum of scores from the four scanned areas.

**Fig 3:**
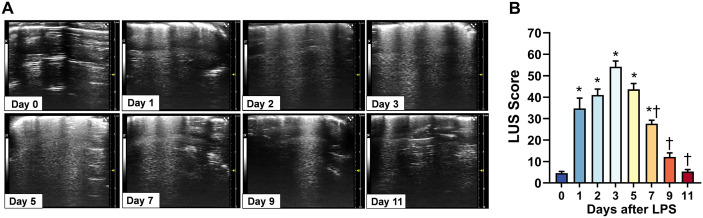
Dynamic changes of lung ultrasound (LUS) imaging and quantitative analysis. **(A)** Representative lung ultrasound images from mice with acute lung injury from 0 (baseline) to 1, 2, 3, 5, 7, 9 and 11 days after tracheal lipopolysaccharide (LPS) instillation, respectively. **(B)** Changes of LUS derived B-line scores over the 11-day study period. *P < 0.05 vs. 0 day, † *P* < 0.05 vs. 3 days, n = 11.

**Fig 4:**
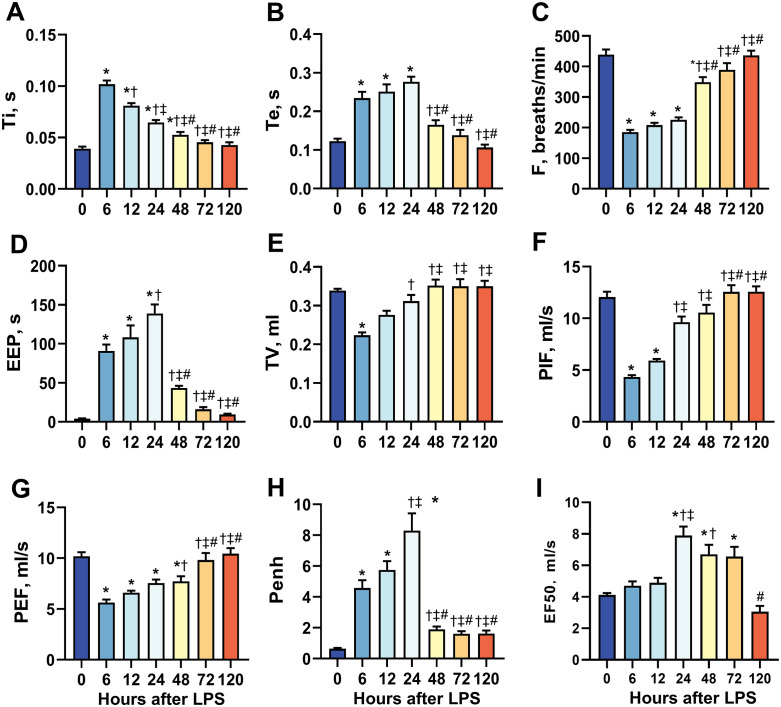
Changes in lung function at 0 (baseline) to 6, 12, 24, 48, 72 and 120 h after LPS stimulation were monitored using WBP. **(A)** Inspiration time (Ti). **(B)** Expiration time (Te). **(C)** Frequency of breathing **(F)**. **(D)** End expiratory pause (EEP). **(E)** Tidal volume (TV). **(F)** Peak inspiratory flow (PIF). **(G)** Peak expiratory flow (PEF). **(H)** Pause enhanced (Penh). **(I)** Expiratory flow at 50% expired volume (EF50). * *P* < 0.05 vs. 0 h † *P* < 0.05 vs. 6 h, ‡ *P* < 0.05 vs. 12 h, # *P* < 0.05 vs. 24 h, n = 11.

Anesthetized animal (5% isoflurane induction followed by 1.5% maintenance) was placed on a heated platform in the supine position. Chest hairs were removed and the four scanning areas were marked (Fig 1A). The scanning sequence was (1) the area between right anterior axillary line and right midclavicular line (Fig 1B); (2) the area between right midclavicular line and anterior median line (Fig 1C); (3) the area between anterior median line and left midclavicular line (Fig 1D) and (4) the area between left midclavicular line and left anterior axillary line (Fig 1E). The scanning was performed with a direction perpendicular to the ribs. LUS images were obtained at the above 4 locations and each scanning session was able to show 4–5 intercostal spaces (Fig 1F).

### 4. B-line score

The scanning images of LUS show two patterns, i.e., A- and B-lines. The A-line is a repetitive horizontal artifact that runs parallel to the pleural line, which is caused by the fact that there is more air than fluid in the lung parenchyma [12]. While the B-line is a linear hypoechoic pattern caused by the ringing effect [16]. The B-line originates from and perpendicularly to the pleural line, radiates deep into the lung field and reaches to the edge of the scanning screen [17]. The B-line is generated by the fact that the sound beam encounters a very thin layer of liquid in its propagation pathway and there is a strong acoustic reflection interface below the liquid [16]. LUS utilizes these principles to detect and quantify the number of B-lines and it derived scores for assessment the severity of pulmonary edema [18]. Dynamic LUS was performed at time points of 0, 1, 2, 3, 5, 7, 9, and 11 days after LPS titration, images across multiple respiratory cycles were recorded and the optimal frame was selected for analysis. Scanning images were analyzed quantitatively using the following methodology to generate corresponding scores based on aforementioned 4 scanning areas (Fig 2A–2D): (1) the presence of only A-lines without B-lines was given a score of zero, indicating the LUS pattern of a normal lung tissue; (2) if B-lines were countable without fusion, a score was given corresponding to the number of B lines, e.g., 2 B-lines equal to score 2; (3) if B-lines were fused and uncountable, the ratio of a B-line width (top) to the length of the corresponding plural line was calculated and multiplied by 10 [15]. A representative ultrasound loop was available in supplementary data (S1 Video). One trained researcher performed lung ultrasound scanning and two researchers analyzed images in a blind fashion to ensure accuracy and reliability of data analysis.

### 5. Lung function test by whole body plethysmography

At the time points of 0, 6, 12, 24, 48, 72, and 120 h post-LPS titration, we assessed pulmonary function in the ALI mice model using the EMMSLink whole-body plethysmography system (EMMS, UK). Monitoring results indicated that pulmonary function in ALI mice returned to normal levels 120 h after LPS treatment, thus this time point was chosen as the final observation. During functional testing, each animal was individually and entirely placed in a separate volumetric plethysmograph chamber of the WBP system without any restraint or sedation. After a 30-min acclimatization period, data were collected at a frequency of once every 2 seconds for a duration of 8–10 min, and average values for each parameter were calculated [19]. The following parameters were analyzed: Inspiratory time (Ti) and expiratory time (Te), representing the duration of inhalation and exhalation within a respiratory cycle, respectively. Respiratory frequency (F) denotes the number of breaths per minute. The end inspiratory pause (EIP) and the end expiratory pause (EEP) refer to the pause time at the end of inhalation before the start of exhalation, or at the end of exhalation before the start of the next inhalation, respectively. Tidal volume (TV) refers to the volume of gas inhaled or exhaled with each breath. Minute volume (MV), the product of respiratory F and TV, reflects the total ventilation per minute [20]. Peak inspiratory flow (PIF) and peak expiratory flow (PEF) represent the highest inspiratory and expiratory flow rates in a breathing cycle, respectively. Together, they reflect airway patency and respiratory muscle strength. The relaxation time (Tr) of the airways, referring to the interval time from the completion of exhalation to the start of the next inhalation. Enhanced pause (Penh) is a sensitive indicator for assessing changes in airway reactivity and resistance. The calculation formula for Penh is: Penh = (Te/Tr)^3^×(PEF/PIF). Expiratory flow at 50% of vital capacity (EF50) is the instantaneous flow rate when half of the exhaled volume has been reached during a forced exhalation, reflecting airway patency [21,22].

### 6. Morphology and lung weights

After LUS examination at designated time-points, mice were anesthetized with 5% isoflurane. Blood was collected from the inferior venous cavity with heparin as the ant-coagulant and plasma was obtained and stored at −80^°^C. Following blood collection, mice were euthanized by increasing the concentration of isoflurane. A midline incision at the ventral part of neck was made to expose the trachea and a transverse incision was made to cut through the trachea. A tracheal cannula was inserted and securely ligated the trachea with the cannula which connected with a 1 ml syringe. Next, thoracic cavity was opened to expose the lung and 1 ml air was slowly injected into the lung to inflate the alveoli. Finally, the lung was resected for morphological examination and weight measurement. A piece (0.5 cm^3^) of left lung was fixed in 10% formalin for histological study. The tibia was collected and the length was measured to normalize the lung weight.

### 7. Histological study

After 4-day fixation, lung tissues were embedded in paraffin blocks and sectioned at a thickness of 5 μm using a Leica RM2125RT slicer (Leica, Germany). Sections were stained with hematoxylin and eosin (H&E) and Sirius Red. Microscopic images were collected by Olympus BX 51 microscope (Olympus Corporation, Japan) and 8–10 views (×20 magnification) from each sample were analyzed. The severity of lung damage was assessed by scoring the area percentage of pulmonary edema, alveolar interstitial inflammation and hemorrhage, and atelectasis observed in each microscopic field of H&E stained tissue sections. The semi-quantitative score was set from 0 to 4 with 0 point for non-injury, 1 point for lesion extent <25%, 2 points for lesion extent 25%−50, 3 points for lesion extent 50%−75%, and 4 points for lesion extent full field of view [6,23,24]. The total score of lung injury was the sum of scores from 8–10 microscopic images. Fibrosis of lung tissue in Sirius red staining was quantified and expressed as an averaged percentage of the Sirius red positive area in the whole lung view at ×20 magnification. Image J was used for data analysis.

### 8. Statistical analysis

Graphpad prism 9.5 software was used for data analysis. All data were expressed as mean ± SEM. One-way ANOVA followed by Tukey post-hoc tests. Correlation analysis between LUS Score and lung injury indices was performed using Pearson’s correlation analysis. *P < 0.05* was considered statistically significant.

## Results

### 1. Dynamic changes of B-lines detected by LUS in murine ALI model

Detection of B-lines by LUS was performed at different stages after ALI induced by LPS stimulation (Fig 3A). The results showed that B-lines began to appear at day 1 after LPS intervention with multiple intercostal spaces presenting single B-lines that had not yet fused or fused B-lines could be observed in only a few intercostal spaces, not in all intercostal spaces. With the prolongation of time, LUS images showed a gradual increase in the number of B-lines and increased fusion. The number of B-lines reached the maximum at day 3, and fused B-lines were observed in almost all intercostal spaces, indicating the development of pulmonary edema. Subsequently, the number of B-lines began to decrease and the fused B-lines gradually separated, and the B-line almost completely disappeared at day 11, the change reflecting that pulmonary edema dissipated (Fig 3A). The LUS derived B-line scores were calculated according to aforementioned method for quantitative analysis to assess the severity of pulmonary edema, and results are showed in Fig 3B.

### 2. Changes of lung function in murine ALI model

Pulmonary function was assessed using the WBP system at 0 (baseline), 6, 12, 24, 48, 72, and 120 h after LPS administration. Both Ti and Te increased sharply (about 1-fold) at 6 h and persisted until 24 h, then quickly returned to baseline levels (Fig 4 A and B). Due to the prolongation of Ti and Te, the total duration of the respiratory cycle increased which led to a decrease in frequency (Fig 4C). EIP and EEP reflect pulmonary ventilation efficiency; EIP remained largely unchanged throughout the process (Table 1), while EEP increased sharply at 6 h and continued to rise until 24 h (approximately 150 times higher than baseline), indicating a obstruction of the airway (Fig 4D). TV decreased by 38% at 6 h after LPS intervention, then gradually returned to baseline levels by 48 h (Fig 4E). The MV, a product of TV × frequency, continued to decrease following LPS intervention, with a reduction of approximately 68% observed at 6 h and then gradually returned to baseline levels after 120 h (Table 1). Both PIF and PEF exhibited similar patterns of change, with both dropping to their lowest levels (50–67% of baseline) at 6 h after intervention and subsequently returning to their baseline levels by 72 h (Fig 4 F and G). Tr, which reflects the severity of obstructive ventilatory disorders, decreased by 30% at 12 h after LPS intervention, reaching its lowest level (49%) at 24–48 h then gradually and partially recovered by 120 h (Table 1). Penh significantly increased (4 times than baseline) at as early as 6 h and peaked (about 8 times higher than baseline) at 24 h then sharply declined by 48 h after LPS intervention (Fig 4H). In contrast, EF50 showed a delayed increase of about 50% above baseline at 24 h, lasting until 72 h. Subsequently, EF50 returns to baseline levels at 120 h post-LPS intervention (Fig 4I).

**Table 1:** Changes in lung function at 0 (baseline) to 6, 12, 24, 48, 72 and 120 h after LPS intervention monitored using WBP.

Variables	0 h (baseline)	6 h	12 h	24 h	48 h	72 h	120 h
Ti (s)	0.04 ± 0.00	0.1 ± 0.00*	0.08 ± 0.00*†	0.06 ± 0.00*†‡	0.05 ± 0.00*†‡#	0.04 ± 0.00†‡#	0.04 ± 0.00†‡#
Te (s)	0.12 ± 0.01	0.23 ± 0.02*	0.25 ± 0.02*	0.28 ± 0.01*	0.17 ± 0.01†‡#	0.14 ± 0.01†‡#	0.11 ± 0.01†‡#
F (breaths/min)	439 ± 13	186 ± 8*	208 ± 7*	225 ± 8*	349 ± 16*†‡#	389 ± 24†‡#	436 ± 16†‡#
EIP (s)	6.13 ± 0.06	6.15 ± 0.05	5.99 ± 0.03	6.01 ± 0.08	6.05 ± 0.03	6.07 ± 0.04	6.23 ± 0.07
EEP (s)	3.9 ± 0.3	90.9 ± 7.7*	107.1.1 ± 16.5*	149.8 ± 19.4*†	43.2 ± 2.5†‡#	13.5 ± 2.5†‡#	9.3 ± 1.0†‡#
TV (ml)	0.34 ± 0.01	0.22 ± 0.01*	0.28 ± 0.01	0.31 ± 0.02†	0.35 ± 0.02†‡	0.35 ± 0.02†‡	0.35 ± 0.01†‡
MV (ml)	126 ± 5	41 ± 2*	56 ± 1*	75 ± 5*†	103 ± 7†‡#	132 ± 10†‡#	150 ± 8†‡#
PIF (ml/s)	12.1 ± 0.4	4.3 ± 0.2*	5.9 ± 0.2*	9.6 ± 0.5†‡	10.6 ± 0.8†‡	12.6 ± 0.7†‡#	12.6 ± 0.5†‡#
PEF (ml/s)	9.9 ± 0.4	5.1 ± 0.3*	6.6 ± 0.1*	7.3 ± 0.4*	7.6 ± 0.5*†	10.0 ± 0.7†‡#	10.8 ± 0.6†‡#
Tr (s)	0.05 ± 0.00	0.06 ± 0.01*	0.06 ± 0.00	0.05 ± 0.00†	0.05 ± 0.00†	0.04 ± 0.00†‡	0.04 ± 0.00†‡
Penh	0.64 ± 0.03	4.57 ± 0.46*	5.75 ± 0.61*	8.30 ± 1.01*†‡	1.90 ± 0.18†‡#	1.61 ± 0.16†‡#	1.62 ± 0.17†‡#
EF50 (ml/s)	4.12 ± 0.11	4.70 ± 0.28	4.90 ± 0.29	7.89 ± 0.58*†‡	6.71 ± 0.66*†	6.58 ± 0.66*	3.06 ± 0.40#

Values are mean ± SEM. LPS, lipopolysaccharide; WBP, whole-body plethysmography; Ti, inspiration time; Te, expiration time; F, frequency of breathing; EIP, end inspiratory pause; EEP, end expiratory pause; TV, tidal volume; MV, minute volume; PIF, peak inspiratory flow; PEF, peak expiratory flow; Tr, relaxation time; Penh, pause enhanced; EF50, expiratory flow at 50% expired volume. * *P* < 0.05 vs. 0 h, † *P* < 0.05 vs. 6 h, ‡ *P* < 0.05 vs. 12 h, # *P* < 0.05 vs. 24 h, n = 11.

### 3. Morphological and weight changes in the lungs of ALI murine

**Fig 5:**
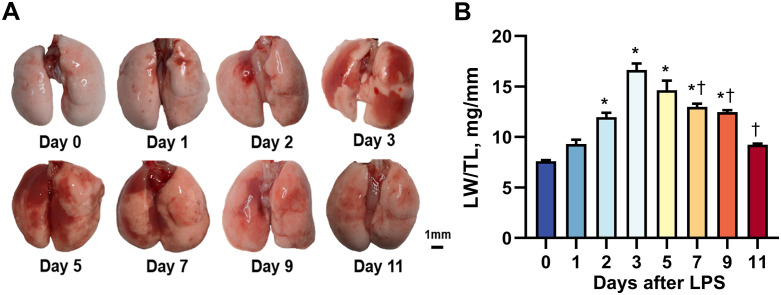
Morphological changes of lungs in mice with acute lung injury. **(A)** Representative photos of lungs from mice at baseline (0) and at day 1, 2, 3, 5, 7, 9 and 11 after LPS stimulation, respectively. The most severe congestion was observed at day 3-5, and basically resolved by day 11. **(B)** Dynamic changes of lung weight/tibial length over the 11 days after LPS intervention. *P < 0.05 vs. 0 day, † *P* < 0.05 vs. 3 days, n = 5-7/group.

The lung was harvested from mice at baseline (day 0) and at day 1, 2, 3, 5, 7 and 11 after LPS intervention for morphological and weight examination. Compared to normal lungs, both the right and left lungs showed patchy congestion, which neared the hilum at day 1–2. At day 3–5 after LPS titration, severe congestion developed presenting as heterogeneous solid lesion expanded to whole lungs with dark red color. From day 7, extent of congestion lesion became less and color of solid lesions got lighter. By day 11, solid lesion (severe congestion) was basically resolved and lung morphology and color gradually back to normal (Fig 5A).

Meanwhile, the lung weight was obtained to assess the severity of pulmonary edema as well. Compared with normal lungs, a significant increase in lung weight normalized by tibia length (LW/TL ratio) was observed at day 2 and the highest level was recorded at day 3 almost being double of normal lung weight after LPS intervention. Subsequently, the LW/TL ratio showed a stepwise decrease from day 5 with the ratio close to the normal lungs by day 11 (Fig 5B). These changes corresponded to the dynamic changes detected by LUS.

### 4. Histological evidences of lung injury in ALI murine

**Fig 6:**
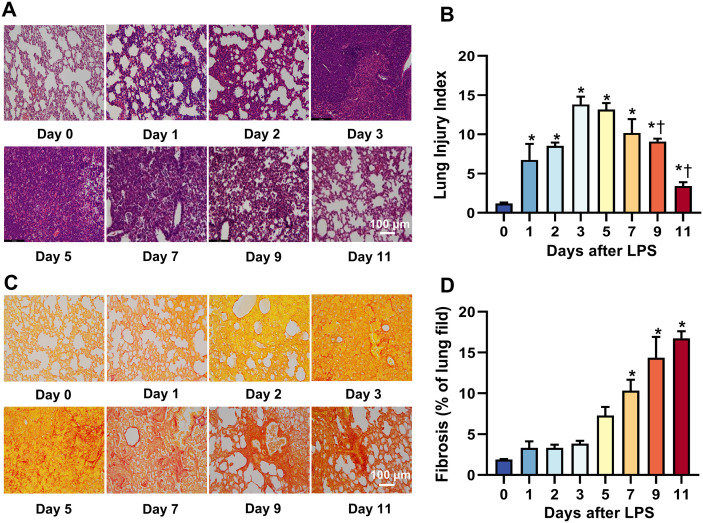
Histological changes of the lung in acute lung injury mice induced by LPS tracheal titration over a 11-day study period. **(A)** Representative microscopic images of the lung with hematoxylin and eosin staining. **(B)** Quantitative analysis of lung injury index. *P < 0.05 vs. 0 day, † *P* < 0.05 vs. 3 days. (**C**) microscopic images of the lung with Sirius red staining. **(D)** Quantitative analysis of pulmonary fibrosis. *P < 0.05 vs. 0 day, n = 5-7/group.

Histological examination was conducted from H&E and Srirus red stained lung sections in mice at different times after LPS intervention. At day 1, inflammatory cells began to recruit to the lungs evidenced by significantly increased nuclei number, alveolar septum thickening, congested blood vessels, pulmonary edema, and the lesion area was < 25%. At day 2, there was a further increased inflammatory cell density and alveolar septa thickening, vascular congestion and pulmonary atelectasis were more pronounced with the lesion area reached to 25%−50%. From days 3–5, a full-field inflammatory cell infiltration was observed, characterized by liver-like solid lesions accompanied by vascular congestion, pulmonary edema, and lung atelectasis. These conditions were at their most severe with the lesion area exceeding 75% and up to 100%. Starting from day 7, the pathological changes observed were less severe compared to those seen between days 3 and 5. However, the damage remained significant, with the lesion area still exceeding 75%. By day 9–11, there was a noticeable regression in inflammatory cell infiltration, vascular congestion and lung atelectasis. However, histological changes remained evident. (Fig 6A). Fig 6B showed a dynamic change of lung injury index derived based on the percentage of pathological area in whole lung view over the 11-day study period.

The extent of fibrosis in lung tissue at different stages after LPS treatment was analyzed by Sirius red staining (collagen fibers in red, lung tissue in yellow). Collagen deposition began to appear around blood vessels at day 3 after LPS treatment, and significant fibrosis could be observed in the whole lung tissue at day 5, and the fibrotic area of the lung tissue gradually increased with time after LPS treatment (Fig 6C and 6D).

### 5. LUS Score correlated with lung weight, lung injury index and lung function parameters

**Fig 7:**
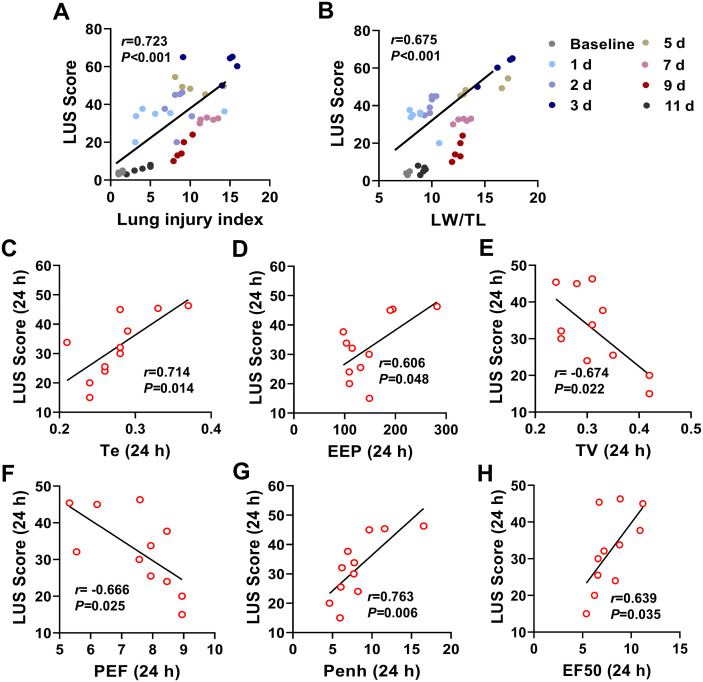
Correlation analysis of lung ultrasound (LUS) scores with the lung injury index, lung weight-to-tibial length ratio (LW/TL), and pulmonary function parameters in a mice model of acute lung injury. LUS scores significantly and positively correlated with the lung injury index (**A**) and the LW/TL ratio (**B**) across all study time points, n = 42. LUS scores positively correlated with expiratory time (Te, **C**) and end-expiratory pause (EEP, **D**) at 24 h after LPS intervention. LUS scores negatively correlated with tidal volume (TV, **E**) and peak expiratory flow (PEF, **F**). LUS scores positively correlated with enhanced pause (Penh, **G**) and expiratory flow at 50% vital capacity (EF50, **H**) at 24 h after LPS intervention. n = 11.

Correlation analyses between LUS Score and lung injury index derived from histological and pathological changes, LW/TL or lung function parameters during the 11-day study period were conducted to further validate the accuracy and reliability of this non-invasive LUS technology in assessment of pulmonary edema. It was found that there was a significant positive correlation between LUS Score and lung injury index (*r* = 0.723, *P* < 0.001), LW/TL (*r* = 0.699, *P* < 0.001) (Fig 7A, 7B). These results demonstrated that LUS Scores are able to dynamically reflect the severity of pulmonary edema in mice with acute lung injury.

To further investigate the relationship between LUS scores and pulmonary function, we analyzed the correlation between LUS scores and pulmonary functional parameters during the study period. Positive correlations between LUS scores and Te, EEP, Penh and EF50 (Fig 7C and 7D, 7G and 7H), while, negative correlations with TV and PEF (Fig 7E and 7F) were observed at 24 h after LPS intervention. Notably, significant correlations between LUS scores and pulmonary function were only observed at the 24 h time-point.

## Discussion

In this study, we modified the intercostal space scanning method and successfully applied it to detect lung injury in mice. Using LUS imaging in the murine ALI model, we observed a dynamic change in B-lines in the intercostal spaces, from a few and uneven distribution at the initial stage to a peak with more and fused B-lines developed, and subsequently, the number and fusion of B-lines began to decrease from day 7–9 after LPS stimulation. The B-line derived LUS scores clearly indicated this progression. These changes reflect the development and dissipation of pulmonary edema following a single exposure to LPS. The application of the LUS technique to the mouse ALI model provides a dynamic and simple method for the quantitative measurement of pulmonary edema.

Currently, LUS has been utilized for detecting pulmonary edema pre-clinic animal models. However, to our knowledge, there are no specific studies targeting the small size mice. In this study, we adapted the previously reported ultrasound techniques in rats by adjusting the ultrasound probe selection to suit the small thoracic cavity and lung structure of mice. We used LUS to assess pulmonary edema in an LPS-induced ALI mouse model and performed quantitative analysis. The LUS scores showed a significant positive correlation with the degree of histopathological lung injury index as well as the lung weight. These findings are consistent with the existing evidence from other animal studies. For instance, Jambrik et al. performed LUS examination during 5–60 min post-injection of oleic acid in minipig ALI model and found a considerable increase in the number of B-lines and the lung wet-to-dry weight ratio with a positive correlation between these two parameters [14]. Similarly, Ma et al [15]. also found a strong correlation between the B-line score and the lung wet weight in non-recovery rat ALI model induced by oleic acid injection. These resultccs demonstrated that LUS technique not only suitable for assessing pulmonary edema in large animals but also meet such application in small size mice. It is worth emphasizing that our study is longitudinal, differing from prior non-recovery ALI models using the LUS technique. Our LPS intratracheal titration model allowed mice to recover and undergo natural course of ALI. Multiple LUS scanning in each mouse precisely tracked pulmonary edema changes. This further underscores the technique’s feasibility in small size animals.

As supportive evidences, morphological and lung weight changes showed a similar trend with most severe lung congestion and the highest lung weight were recorded at day 3–5. Elevated lung weight is the hard evidence of pulmonary edema, impressively, there was a signficant positive correlation between LUS scores and lung weight. In addition, we also examined pathological damage from lung histological sections, inflammatory cells infiltration, thickening of alveolar septa, vascular congestion and pulmonary atelectasis emerged at day 1, and reached to the maximum at day 3–5 and then slowly regressed thereafter. The lesion area derived dynamic lung injury index also had a significant positive correlation with LUS score. These correlations validated the accuracy and reliability of LUS technique in assessment the severity of pulmonary edema and the degree of lung injury [21].

In this study, we also measured pulmonary function within 5 days following LPS attack. A significant prolongation of Ti and Te led to a decrease in respiratory frequency, indicating a decline of general respiratory state. Both EIP and EEP reflect pulmonary ventilation efficiency, EEP showed a sharp increase as early as 6 h and then declined after 24 h, while EIP did not change. An increased EEP could suggest issues with the respiratory cycle such as air trapping or difficulty initiating the next breath, which might be seen in conditions like chronic obstructive pulmonary disease. As Ti, Te, EIP and EEP are the key factors that consist of one whole respiratory cycle, prolonged Ti, Te and EEP did not lead to a rise in TV, in contrast, TV exhibited a decrease at 6 h, indicating impaired alveolar compliance. In ALI, the inflammatory response within the alveoli and interstitium causes thickening of the alveolar walls and interstitial edema owing to increased capillary permeability. Moreover, accumulation of extravascular lung water results in a disruption of alveolar surfactant. These factors collectively contribute to a decrease in the stability and compliance of the alveoli, increasing their susceptibility to collapse. This leads to a reduction in the effective gas exchange area and an increase in respiratory resistance, making it more difficult for air to enter the alveoli. The interplay of these factors ultimately results in a decrease in tidal volume [25]. In addition, both PIF and PEF displayed a similar decline at 6 h and recovered progressively, suggesting the presence of airway obstruction, increased airway resistance and damage of the mechanics of breathing at the early phase of LPS intervention. Penh and EF50 are distinct respiratory function parameters that provide insights into airway resistance and obstruction, particularly in small airways. Penh, a dimensionless parameter primarily reflecting respiratory patterns [26,27], shows a sharp increase at 6 h post-LPS intervention, lasting only up to 24 h. This transient response may indicate a rapid airway reaction to the direct stimulation by LPS. In contrast, EF50, a physiological variable measured in milliliters per second (ml/s), rises at 24 h and continues until 72 h after LPS intervention, suggesting a more sustained response associated with the progression of airway obstruction induced by pulmonary edema. While Penh’s validity as a reliable measure of airway mechanics is questioned due to its sensitivity to non-pulmonary mechanics factors such as humidification, warming of inhaled gases, hyperoxia, and ventilation timing. EF50 is considered a more reliable method for measuring airway resistance. It is not influenced by changes in respiratory frequency, exhalation time, or tidal volume, offering a more direct and accurate assessment of airway resistance. In essence, EF50 is more indicative of airway resistance, whereas Penh reflects changes in respiratory patterns to a greater extent [22,28]. However, Penh is a widely used index of pulmonary function in asthma model research, known for its sensitivity in reflecting the degree of airway constriction [29]. Previous studies have shown that there is a significant positive correlation between changes in Penh and pulmonary resistance parameters. This correlation indicates that an increase in Penh is typically associated with enhanced reactivity of the airways to stimuli, and airway hyperresponsiveness is a core pathological feature of asthma. Therefore, as a quantitative indicator, Penh not only describes the state of airway constriction but also aids in assessing the reactivity of airways to stimuli in asthma models [30]. All of these aberrant changes tended to be recovered at 72 h after LPS attack that possibly indicated a short adverse influence in pulmonary function by a single LPS attack, a continuous inflammatory stimulation may generate a long-lasting functional damage which require a further investigation. During the study period, we multiply measured pulmonary function by WBP, and found LUS scores only correlated with a number of pulmonary functional indices obtained at 24 h after LPS intervention.

A striking finding from this study was non-synchronization between alterations of pulmonary function and histology induced by a single tracheal LPS attack. This suggests that the dynamic changes in pulmonary function preceded histological alterations. A similar finding by Rojas et al reported an early alteration of pulmonary function at 4–6 h following intraperitoneal injection of LPS in mice and recovered at 48 h [19]. Another study investigated pulmonary function in a murine ALI model induced by aerosolized LPS inhalation and observed that respiratory parameters changed most significantly at 48 h and had returned to baseline levels by 96 h [31]. Nevertheless, our study covered a broader time range, thereby more accurately reflecting the dynamic changes. The methods used for establishing ALI models and the timing for pulmonary function test can significantly influence the results. Pulmonary function tests can rapidly reflect physiological changes in the lungs, such as the ventilatory capacity of alveoli and the efficiency of gas exchange. These functional changes can be detected before structural damage occurs because they are closely related to inflammatory responses and changes in the permeability of the alveolar-capillary membrane [32]. Therefore, changes in pulmonary functional indices can serve as early indicators of lung inflammation and injury. However, the discrepancy between functional and histological changes may highlight the limitations of WBP as a non-invasive diagnostic tool for pulmonary function testing. As Glaab pointed out in a recent review [22], there is currently no gold standard for measuring pulmonary function in mice, as no single available invasive or non-invasive method is optimal in all respects. There is a need to develop more sensitive and comprehensive diagnostic methods to accurately assess lung health and track disease progression.

The inflammatory response triggered by LPS causes the accumulation of inflammatory cells in the alveoli and interstitium, disrupting the alveolar structure and leading to the necrosis of alveolar epithelial cells. This persistent inflammatory reaction and tissue damage further activate alveolar fibroblasts, prompting their abnormal proliferation and the production of large amounts of extracellular matrix, particularly collagen, ultimately leading to the development of pulmonary fibrosis. In the LPS-induced ALI model, the repair and remodeling of lung tissue involve a complex interplay among alveolar epithelial cells, endothelial cells, and fibroblasts. These cells participate in the repair of alveolar and vascular structures and the regulation of inflammation by secreting various bioactive molecules, thereby affecting the recovery of lung function and the progression of fibrosis [33,34]. Since the lungs are the only internal organs in constant contact with the external environment through respiration, they are particularly susceptible to various infections and toxins, which can lead to the occurrence of ALI [35]. In some cases, ALI may further deteriorate into permanent pulmonary fibrosis, as seen in lung damage caused by the novel coronavirus. Data from the COVID-19 pandemic indicate that lung damage caused by SARS-CoV-2 infection can lead to severe fibrosis. Many COVID-19 patients exhibit a reticular pattern of pulmonary fibrosis on CT scans and suffer from long-term lung function impairment [36]. In patients with severe pulmonary fibrosis, the forced vital capacity and diffusing capacity for carbon monoxide are significantly reduced due to the destruction of lung tissue structure and the decline in lung function. The decline in these indicators not only reflects the degree of lung parenchymal damage but also implies a reduced efficiency of oxygen and carbon dioxide exchange between the alveoli and the blood. Consequently, patients experience dyspnea, decreased activity tolerance, and a general deterioration in health status, severely affecting their quality of life [37]. In our study, a noteworthy finding was that even a single LPS attack could lead to a long-lasting fibrosis. Moreover, despite most pathological changes having resolved, the degree of fibrosis could still increase over time following LPS intervention.

In summary, by systemic investigating LUS technique in assessment the progression of pulmonary edema in mice ALI model induced by LPS tracheal intervention, our results document the usefulness, accuracy and reliability of this non-invasive technique. LUS provides a simple, quantitative mean for dynamic monitoring pulmonary edema which corresponding to lung pathological changes in small size animals.

## Supporting information

S1 Video A cycle of lung ultrasound (Video). (ZIP)

S2 File Raw data. (XLSX)
